# Respiratory Physiotherapy Techniques in Neonatal Intensive Care Units: A Scoping Review

**DOI:** 10.1002/pri.70304

**Published:** 2026-08-01

**Authors:** Nilson Willamy Bastos de Souza Júnior, Ana Tereza do Nascimento Sales Figueiredo Fernandes, Karolinne Souza Monteiro, Adynna Tévina de Castro Silva, Adriele de Morais Nunes, Josiane Marques Felcar, Silvana Alves Pereira

**Affiliations:** ^1^ Postgraduation Program in Rehabilitation Sciences UEL‐UNOPAR State University of Londrina (UEL) Londrina Paraíba Brazil; ^2^ Department of Physiotherapy Paraíba State University (UEPB) Campina Grande Paraíba Brazil; ^3^ Department of Physiotherapy Federal University of Rio Grande do Norte (UFRN) Rio Grande do Norte Brazil; ^4^ Multiprofessional Residency in Neonatal Intensive Care Federal University of Rio Grande do Norte UFRN Rio Grande do Norte Brazil; ^5^ Postgraduatoin Program in Rehabilitation Sciences at the Federal University of Rio Grande do Norte (UFRN) Federal University of Rio Grande do Norte UFRN Rio Grande do Norte Brazil

**Keywords:** infant, newborn, physical therapy modalities, respiratory mechanics, respiratory therapy

## Abstract

**Background and Purpose:**

Certain physiological characteristics increase the susceptibility of newborns, particularly preterm infants, to pulmonary collapse. In addition, newborns are prone to the accumulation of airway secretions, which, even in small volumes, can compromise respiratory function. Consequently, breathing therapy plays a crucial role in promoting airway clearance and supporting effective ventilation in this population. This scoping review aimed to map the breathing therapy methods used for newborns admitted to intensive care units and also to identify the clinical outcomes assessed in the studies.

**Methods:**

We conducted a scoping review of all clinical studies involving hospitalized newborns of both sexes aged 0–28 days who received conventional or nonconventional respiratory physiotherapy. Exclusion criteria were studies on animals, reviews, protocols, or studies that were unavailable or incomplete. Outcomes analyzed included respiratory distress, cardiorespiratory parameters, thoracoabdominal synchrony, pain, and lung volumes and capacities.

**Results:**

The search yielded 1261 articles. Twelve studies were included for data extraction and synthesis. The main respiratory techniques studied were vibration, percussion, postural drainage, increased expiratory flow, lung squeezing (pulmonary hyperinflation using a manual resuscitator), and muscle synergy techniques.

**Discussion:**

The included studies reported short‐term changes in cardiorespiratory parameters following the interventions, while no consistent evidence of increased pain was observed. However, the available evidence is heterogeneous and predominantly derived from single‐session interventions, highlighting the need for well‐designed and adequately powered studies to further evaluate the efficacy and safety of these interventions in this population.

## 1 Introduction

Mechanical ventilation and respiratory support are frequently required in hospitalized neonates due to their distinct anatomophysiological characteristics, such as immature and underdeveloped respiratory muscles, a more horizontal rib orientation, and reduced alveolar caliber, all of which predispose them to respiratory failure (A. Nunes et al. 2022). Approximately 52% of newborns admitted to neonatal intensive care units required mechanical ventilation at some point during their hospitalization (Sauthier et al. 2021).

Furthermore, specific physiological factors (such as weakness of the respiratory musculature, a more distensible rib cage resulting from the maturational deficit, and an irregular respiratory rhythm) render newborns particularly susceptible to alveolar collapse. Neonates are also more prone to mucus accumulation, even in small amounts, which can significantly increase airway resistance and reduce airflow (Sauthier et al. 2021; Rocha et al. 2018). In this context, respiratory physiotherapy techniques play a crucial role, as they can markedly influence respiratory biomechanics (Ribeiro et al. 2020). These techniques enhance ventilation by promoting the re‐expansion of collapsed lung areas (J. X. Santos et al. 2023) and facilitating the clearance of secretions (De Souza et al. 2023), thus helping to maintain airway patency in these vulnerable patients (Rocha et al. 2018; Ribeiro et al. 2020; J. X. Santos et al. 2023; De Souza et al. 2023; De Abreu et al. 2011).

Respiratory physiotherapy involves applying physical interventions, tailored to individual anatomical and physiological features, pulmonary pathology, and related clinical conditions. The main goals are to optimize gas exchange and reduce the work of breathing (Hough et al. 2008). In neonates, techniques include positioning, increased expiratory flow, prolonged slow expiration, and lung expansion maneuvers. Each has specific indications and contraindications (Johnston et al. 2012). These techniques are often combined, making it hard to determine their individual effects. There is also no consensus on the best technique or benefits for neonates (Johnston et al. 2012).

Given this context, this scoping review aimed to map the respiratory physiotherapy techniques described for neonates admitted to neonatal intensive care units. It also aimed to identify the clinical outcomes assessed in the literature.

## 2 Methods

### 2.1 Study Design

This scoping review was conducted in accordance with the methodological recommendations of the Joanna Briggs Institute (Peters et al. 2015) and reported following the PRISMA‐ScR guidelines (Tricco et al. 2018). A prior protocol was developed and registered on the Open Science Framework platform (doi:10.29390/001c.140878) (A. M. Nunes et al. 2025). Ethical approval is not required for the article type.

### 2.2 Eligibility Criteria

Clinical studies evaluating respiratory physiotherapy interventions in neonates admitted to neonatal intensive care units were considered eligible, including randomized controlled trials and non‐randomized interventional studies. These studies were included to enable the mapping of respiratory physiotherapy techniques used in neonatal care and the outcomes reported in clinical settings. No restrictions were imposed regarding intervention protocols, comparator groups, or outcome measures. There were no restrictions regarding publication year or language.

The study population comprised neonates of either sex aged 0–28 days. Studies involving neonates with congenital heart disease, genetic syndromes, or thoracic deformities were excluded. Animal studies, review articles, study protocols, and studies for which the full text was unavailable were also excluded.

### 2.3 Interventions

For this review, studies examining the use of conventional or non‐conventional respiratory physiotherapy techniques in newborns hospitalized in Neonatal Intensive Care Units were considered. Conventional techniques included vibration maneuvers, percussion, and postural drainage, while non‐conventional techniques included increased expiratory flow, autogenic drainage, lung squeezing (hyperinflation assisted by a manual resuscitator), and techniques focusing on muscle synergy (Gomes 2016).

### 2.4 Outcomes

Outcomes reported by the included studies were extracted and grouped into the following domains: (a) respiratory distress: assessed using any instrument specifically designed for this purpose (e.g., the Silverman‐Andersen score); (b) cardiorespiratory parameters: heart rate (HR) and peripheral oxygen saturation (SpO2),both measured by multiparameter monitors, and respiratory rate (RR), assessed by counting respiratory excursions over 1 minute; (c) thoracoabdominal synchrony: evaluated either by observer assessment or by instruments capable of detecting thoracoabdominal movement (e.g., biophotogrammetry or plethysmography); (d) pain: assessed using specific scales validated for the population under study; (e) lung volumes and capacities: evaluated using a pneumotachograph or flow transducer.

#### 2.4.1 Search Strategy

A comprehensive literature search was conducted in PubMed, Cochrane Library, LILACS, Web of Science, SciELO, and Science Direct from database inception to January 2026, without language restrictions. The search strategy combined terms related to neonates, respiratory physiotherapy techniques, and clinical studies evaluating these interventions. Indexed search terms from DeCS/MeSH were used and then combined with the Boolean operators AND OR (Table 1). More details about the descriptors used are provided in Table 1.

**TABLE 1:** Descriptors and keywords used for searching on databases.

	Mesh term	Keywords
P—population	Newborn	Newborn OR newborns OR newborn infant OR newborn infants OR neonate OR neonates
C—concept	Physical therapy modalities, physical therapy Specialty, respiratory therapy	Chest physical therapy OR conventional chest physical therapy OR chest physiotherapy OR physiotherapy respiratory OR physiotherapy OR autogenic drainage OR expiratory flow increase technique OR prolonged slow expiratory maneuver OR technique of Re‐educating thoracic and abdominal OR chest percussion OR Shaking OR Tapping
C—context	Clinical trials as topic random allocation therapeutic use	Clinical trials as topic OR clinical trial as topic OR clinical trial OR random OR random allocation OR randomization OR therapeutic use OR therapeutic uses OR therapeutic effects OR therapeutic effect OR interventional study OR interventional studies

The strategy was adapted as appropriate for the syntax and indexing system of each database. The complete search strategies for all databases are provided in Supporting Information S1.

#### 2.4.2 Study Selection and Data Extraction

The selection and evaluation of the studies occurred in four stages:

Stage 1: Search in the PubMed, Cochrane Library, LILACS, Web of Science, SciELO, and Science Direct databases using the main strategy and its variations.

Stage 2: Duplicate records were removed—All references records from the database search were uploaded into the reference management software Rayyan QCRI (Ouzzani et al. 2016) (https://rayyan.qcri.org), where duplicate studies were initially excluded.

Stage 3: Title and Abstract Screening—Two independent reviewers (NWBSJ and ATNSFF) conducted this step. In cases of disagreement, a third independent reviewer (JMF) reviewed the article and then confirmed or rejected the inclusion of the specific publication. All reviewers conducted the screening according to the eligibility criteria for article selection.

Stage 4: Full‐text articles were independently assessed by two reviewers. For studies meeting the eligibility criteria, data extraction was independently performed by the same reviewers using a standardized data extraction form. The extracted information included bibliographic characteristics (title, authors, and year of publication), study setting, study design, objectives, respiratory physiotherapy techniques evaluated, outcomes assessed, and main conclusions. Any disagreements arising during study selection or data extraction were resolved through consultation with a third reviewer (JMF).

#### 2.4.3 Data Synthesis

The characteristics of the interventions, as well as categorical variables, were synthesized descriptively and presented in tables. Numerical variable results extracted from the articles were presented as means and standard deviations or medians and interquartile ranges. The extracted data were synthesized descriptively according to the objectives of the scoping review. Studies were grouped according to respiratory physiotherapy technique, neonatal population, and reported outcome domains. No meta‐analysis or quantitative synthesis was performed due to the methodological and clinical heterogeneity of the included studies.

### 2.5 Results

#### 2.5.1 Descriptive Mapping of Populations and Interventions

**FIGURE 1:**
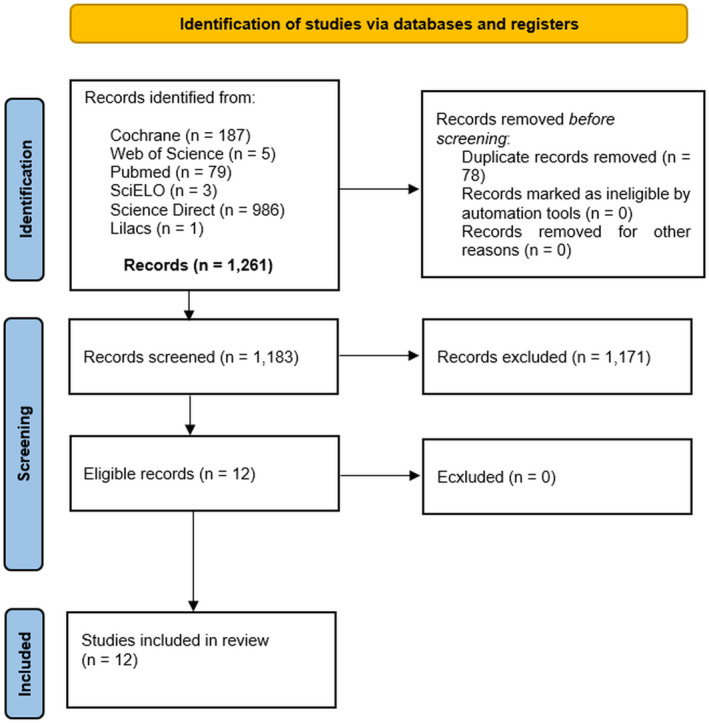
PRISMA flow diagram for new systematic reviews which included searches of databases and registers only.

The search returned 1261 articles. After duplicate removal (*n* = 78), titles and abstracts were screened for eligibility, and potentially relevant articles were assessed through full‐text review. Following the analysis of the eligibility criteria for this review, 12 studies met the criteria and were included in the final synthesis. More detailed information on article selection is provided in Figure 1.

Among the 12 included studies, 7 were randomized clinical trials, 1 was a cross over non‐randomized clinical trial, 1 was a cross‐sectional study, 1 was an experimental study and 2 were prospective cohort studies. Publication years ranged from 2003 to 2021, with most studies conducted in Brazil.

Most studies included preterm neonates receiving respiratory support, although some investigated healthy neonates or infants with transient tachypnea, pneumonia, respiratory distress syndrome, or atelectasis. Further details on the newborns' demographic and clinical descriptions are available in Table 2.

**TABLE 2:** Characteristics of the participants.

Author, year	Sample (EG/CG)	Male (%)	Age (days)	GA (weeks)	Weight (g)
			M ± SD/Md (IQR)		
Oliveira et al. (2021)	EG: 29	EG: 51.7	EG: 6 [2.5–15]	EG: 38 [36.8–40]	EG: 2990 [2510–3505]
	CG: 20	CG: 45	CG: 3 [2–6]	CG: 38.2 [35.7–39]	CG: 2850 [2500–3172]
Diwate et al. (2018)	EG: 6	EG: 33.3	EG: 1.5 ± 0.5	NR	EG: 1333 ± 152.8
	CG: 6	CG: 50	CG: 1.83 ± 0.37		CG: 1316.6 ± 111.9
Guerra et al. (2017)	EG: 20	EG: 40	EG: 1 [0–2]	EG: 39 [37–41]	EG1: 3345 [2540–3930]
Martins et al. (2013)	EG1: 20	EG1: 40	EG1: 10.75 ± 6.9	EG1: 31.25 ± 2.36	EG1: 1471.5 ± 414.98
	EG2: 20	EG2: 60	EG2: 15.15 ± 7.68	EG2: 30.75 ± 2.88	EG2: 1655.25 ± 486.01
	CG: 20	CG: 30	CG: 13.75 ± 7.17	CG: 31 ± 1.92	CG: 1683.5 ± 403.19
Mehta et al. (2016)	60	70	9.55 ± 5.86	NR	1550 ± 511.5
Moura et al. (2017)	EG1: 14	EG1: 64.29	NR	EG1: 33.5 ± 2.65	EG1: 609.43 ± 376.25
	EG2: 14	EG2: 57.14		EG2: 33.07 ± 2.84	EG2: 1550.36 ± 424.09
Nicolau and Falcão (2010)	42	43	NR	29.5 ± 2.1	1024 ± 281
Oliveira et al. (2017)	EG1: 20	51	NR	38	2940
	EG2: 29				
M. L. M. Santos et al. (2009)	EG1: 9	EG1: 66.6	EG1: 4.0 ± 1.48	EG1: 31.1 ± 2.26	EG1: 1.257.8 ± 237.4
	EG2: 9	EG2: 55.5	EG2: 9.0 ± 6.6	EG2: 30.8 ± 1.48	EG2: 1.202.8 ± 194.3
Tavares et al. (2019)	30	50	1.5 ± 0.9	30.2 ± 3	990.5 [790.5–1365]
Wong and Fok (2003)	EG1: 26	EG1: 46.2	NR	EG1: 27.9 ± 2.9	EG1: 1042 ± 472
	EG2: 30	EG2: 66.7		EG2: 28.3 ± 3.7	EG2: 1036 ± 469
Wong and Fok (2006)	11	NR	NR	29.7 ± 2.3	1258 ± 419

With regard to the intervention protocols, substantial heterogeneity was observed across the included studies. Most investigations (Oliveira et al. 2021; Guerra et al. 2017; Martins et al. 2013; Mehta et al. 2016; Oliveira et al. 2017; M. L. M. Santos et al. 2009; Tavares et al. 2019; Wong and Fok 2006) evaluated the effects of a single respiratory physiotherapy session, whereas only a minority assessed repeated sessions over consecutive days. In addition, several intervention protocols combined two or more respiratory physiotherapy techniques rather than evaluating isolated maneuvers, limiting comparisons across studies.

Conventional respiratory physiotherapy techniques were the most frequently investigated (Oliveira et al. 2021; Diwate et al. 2018; Martins et al. 2013; Mehta et al. 2016; Moura et al. 2017; Nicolau and Falcão 2010; Oliveira et al. 2017; M. L. M. Santos et al. 2009; Wong and Fok 2003, 2006), including vibration (or vibrocompression), percussion, postural drainage, and lung squeezing (manual hyperinflation using a manual resuscitator). Less frequently evaluated interventions (Guerra et al. 2017; Moura et al. 2017; Nicolau and Falcão 2010; Oliveira et al. 2017; Tavares et al. 2019) included increased expiratory flow (forced expiratory flow acceleration) and muscle synergy–based techniques (Thoracoabdominal Rebalancing–TAR). Detailed intervention characteristics are presented in Table 3.

**TABLE 3:** Characteristics of the interventions.

Author, year	Intervention type	Data collection tools and valuation	Protocol characteristics	*N* sessions per day and treatment time	Pathologies
Oliveira et al. (2021)	Techniques focusing on muscle synergy; or “conventional” respiratory therapy (expiratory passive manual therapy, expiratory vibrocompression and prolonged slow expiration technique).	Neonatal infant pain scale evaluation in a calm ambient, during a 5‐min period immediately after the intervention.	EG: Techniques focusing on muscle synergy; protocol: Inferior abdominal support, thoraco‐abdominal support, ileo‐costal support, and inspiratory aid.	1x, single session	NA
		Monitoring of heart rate, respiratory rate, peripheral oxygen saturation, axillary temperature, were assessed before and after the intervention.	CG: Conventional chest respiratory protocol: Manual passive expiratory therapy, chest expiratory vibration, chest expiratory compression, chest vibration or prolonged slow expiration.		
Diwate et al. (2018)	Percussion (using a chest percussion cup, hands or fingers), vibration and prone position	Monitoring of saturation of peripheral oxygen, partial pressure of arterial oxygen and peak inspiratory pressure.	EG: Conventional chest therapy (percussion‐including cupping with face mask, contact heel percussion, and finger percussion and vibration with fingers) + prone position.	2x, for 3 days	NA
			CG: Only conventional chest therapy.		
Guerra et al. (2017)	Expiratory flow acceleration.	Assessment of thoracoabdominal area by photogrammetry before and after intervention.	EG: Expiratory flow acceleration technique performed for 5 minutes with a 15‐s interval between them.	1x, single session	NA
Martins et al. (2013)	Conventional respiratory therapy (vibration using a mechanical vibrator device along with gentle chest compression) and techniques focusing on muscle synergy (thoracoabdominal support, lower abdominal support, lateral costal support and chest mobilization).	Monitoring of cardiorespiratory parameters, pulse oximetry, pulmonary auscultation; neonatal infant pain scale; neonatal facial coding system; premature infant pain profile.	EG1: Mechanical or manual vibrocompression, 20 min.	1x, single session	NA
			EG2: 4 × 5 min of techniques focusing on muscle synergy (thoracoabdominal support, lower abdominal support, lateral costal support and chest mobilization), total of 20 min.		
			CG: Rest.		
Mehta et al. (2016)	Postural drainage, thoracic percussion, vibration and airway suctioning.	Monitoring of cardiorespiratory parameters, saturation of peripheral oxygen, pulmonary auscultation, chest x‐ray and Silverman‐Andersen score.	EG: Positioning for postural drainage for 10 minutes. Percussions and vibrations were given intermittently during postural drainage; airway suctioning if necessary.	1x, single session	NA
Moura et al. (2017)	Vibration and expiratory flow acceleration.	Evaluation of pain by the premature.	The techniques in both groups were carried out with the infants positioned in dorsal decubitus, at the time of the expiration phase, with a maximum duration of 10 minutes.	1x a day, during 3 days	Pneumonia
		Infant pain profile at three times; monitoring of cardiorespiratory parameters, pulse oximetry.			
Nicolau and Falcão (2010)	Postural drainage along with manual vibration, diaphragmatic and ribs support, airway suctioning.	Monitoring of cardiopulmonary parameters (heart rate, blood pressure, pulse oximetry).	Positioning in right and/or left decubitus along with manual vibration, diaphragmatic and ribs support, and airway suction (if necessary).	2–3x/day. Minimum of 6 sessions	NA
Oliveira et al. (2017)	Conventional respiratory therapy (expiratory passive manual therapy, expiratory chest compression, chest vibration or prolonged slow expiration technique) and techniques focusing on muscle synergy.	Monitoring of cardiopulmonary parameters (heart rate, respiratory rate pulse oximetry); axillary temperature; pain evaluation by neonatal infant pain scale; respiratory distress by Silverman‐Andersen score and Downes' score.	EG1: 15‐min conventional respiratory therapy session (expiratory passive manual therapy, expiratory chest compression, chest vibration or prolonged slow expiration technique).	1x, single session	Transient tachypnea of the newborn
			EG2: 15‐min of techniques focusing on muscle synergy (lower abdominal support, thoracoabdominal support, lateral‐costal support and inspiratory aid.		
M. L. M. Santos et al. (2009)	Expiratory chest compression and airway suctioning.	Monitoring of cardiopulmonary parameters (heart rate, pulse oximetry); airway resistance and dynamic compliance by pneumotachograph.	Association of therapeutic positioning along with manual expiratory chest compression and airway suctioning	1x, single session	NA
Tavares et al. (2019)	Chest vibration along with: Expiratory passive manual therapy and diaphragmatic stimulation technique, airway suctioning.	Monitoring of cardiopulmonary parameters (heart rate, pulse oximetry, respiratory rate); axillary temperature; pain evaluation by neonatal infant pain scale and neonatal Facial coding system scales; visual observation of chest retractions presence.	15‐min conventional respiratory therapy session: Positioning in both lateral left and right decubitus along with chest vibration and: Expiratory passive manual therapy technique. Afterward, diaphragmatic stimulation technique in supine position. Airway suction, if necessary.	1x, single session	Respiratory distress syndrome
Wong and Fok (2003)	Lung squeezing, postural drainage, percussion and vibration. Airway suctioning if necessary.	Evaluation of atelectasis presence by chest radiography; number of sessions necessary for lung expansion; presence of pulmonary secretions by pulmonary auscultation; monitoring of cardiopulmonary parameters	EG1: 10‐min session of “lung squeezes” which consisted of 3–4 sustained chest compressions lasting for about 5 seconds, followed by a gentle slow “release phase”.	2x/day, during 3 days minimum	Preterm neonates with atelectasis
			EG2: 10‐min session of chest percussion and vibration techniques in modified postural drainage positions.		
Wong and Fok (2006)	Lung squeezing (3–5 chest compressions with a 5 second duration followed by gentle and slow release).	Evaluation of respiratory system compliance and respiratory system resistance by pneumotachograph	10‐min session of “lung squeezes” which consisted of 3–4 sustained chest compressions lasting for about 5 seconds, followed by a gentle slow “release phase”.	1x, single session	Respiratory distress syndrome

#### 2.5.2 Reported Outcomes and Physiological Responses

Due to the considerable clinical and methodological heterogeneity across the included studies, the reported outcomes varied substantially. The extracted outcomes were grouped into five domains: cardiorespiratory parameters (9/12 studies), pain (5/12 studies), respiratory distress (3/12 studies), lung mechanics (2/12 studies), and thoracoabdominal synchrony (1/12 study). Overall, the included studies primarily reported immediate physiological responses following respiratory physiotherapy interventions, whereas clinically relevant longer‐term outcomes were rarely assessed. Most outcomes were assessed immediately after single‐session interventions, with very limited evaluation of repeated treatments or follow‐up beyond the intervention period.

Most studies evaluated cardiorespiratory parameters immediately after the intervention. Authors reported short‐term changes in heart rate (Mehta et al. 2016; Moura et al. 2017; Nicolau and Falcão 2010; Tavares et al. 2019), respiratory rate (Oliveira et al. 2021; Mehta et al. 2016), and peripheral oxygen saturation (Oliveira et al. 2021; Diwate et al. 2018; Mehta et al. 2016; Nicolau and Falcão 2010). A summary of these findings is presented in Table 4.

**TABLE 4:** Outcome characteristics in cardiorespiratory parameters.

Author, year	HR (bpm)	*p*‐value	RR (cpm)	*p*‐value	SpO2 (%)	*p*‐value
Oliveira et al. (2021)	EG (baseline): 138 [129–148]	0.67	EG (baseline): 74 [68–76]	0.07	EG (baseline): 97 [95–98]	0.16
	CG (baseline): 136 [131–142]		CG (baseline): 67 [65–70]		CG (baseline): 95 [93–97]	
	Before intervention: 137 [130–145]	0.46	Before intervention: 70 [66–75]	< 0.001	Before intervention: 96 [94–98]	0.003*
	After intervention: 136 [128–146]		After intervention: 58 [55–65]		After intervention: 98 [96–99]	
Diwate et al. (2018)	NR	—	NR	—	EG: 96.20 ± 0.84	0.02*
					CG: 93.20 ± 2.28	
Guerra et al. (2017)	NR	—	NR	—	NR	—
Martins et al. (2013)	EG1:	0.949 0.073 0.311	EG1:	0.117 0.428 0.618	EG1:	0.120 0.759 0.256
	T1: 148.60 ± 12.39		T1 57.70 ± 7.29		T1: 96.30 ± 1.8	
	T2: 146.95 ± 15.8		T2: 57.35 ± 7.15		T2: 96.85 ± 1.57	
	T3: 146.05 ± 13.70		T3: 55.30 ± 7		T3: 96.40 ± 1.70	
	EG2:		EG2:		EG2	
	T1: 153.15 ± 14.63		T1: 56.10 ± 10.50		T1: 95.70 ± 2.56	
	T2: 147.25 ± 16.25		T2: 58 ± 8.29		T2: 95.85 ± 2.46	
	T3: 150.40 ± 15		T3: 57.20 ± 11.18		T3: 95.90 ± 2.00	
	CG:		CG:		CG:	
	T1: 147.45 ± 16.50		T1: 55.75 ± 7.75		T1: 95.65 ± 2.54	
	T2: 149.20 ± 14.63		T2: 57.50 ± 6.56		T2: 96.05 ± 2.63	
	T3: 152.10 ± 14.15		T3: 56 ± 8.47		T3: 96 ± 2.25	
Mehta et al. (2016)^a^	Significant decrease after 15 minutes of intervention.	0.01*	Significant decrease was after 15 minutes of intervention.	< 0.0001*	Significant increase after 15 minutes of intervention.	< 0.0001*
Moura et al. (2017) values represent mean change from baseline	Ec:	0.023* 0.358	NR	—	Ec:	0.76 0.00
	Day 1: 0.36 ± 0.84				Day 1: 0.29 ± 0.83	
	Day 2: 0.43 ± 0.51				Day 2: 0.07 ± 0.27	
	Day 3: 0.07 ± 0.27				Day 3: 0.14 ± 0.36	
	CG:				CG:	
	Day 1: 0.07 ± 0.27				Day 1: 0.00 ± 0.0	
	Day 2: 0.14 ± 0.51				Day 2: 0.00 ± 0.0	
	Day 3: 0.00 ± 0.0				Day 3: 0.00 ± 0.0	
Nicolau and Falcão (2010)	Baseline: 155 [160–150]	< 0.001*	Baseline: 51 [50–52]	No significant difference	Baseline: 92 [89–95]	< 0.001*
	After intervention: 152 [146–156]		After intervention: 49 [48–50]		After intervention: 96 [92–98]	
Oliveira et al. (2017)	EG1 (baseline): 136 [109–171]	0.67 0.82	EG1 (baseline): 69 [60–90]	0.06 0.18	EG1 (baseline): 95 [83–100]	0.16 0.74
	EG2 (baseline): 139 [120–161]		EG2 (baseline): 73 [63–90]		EG2 (baseline): 96 [90–100]	
	EG1 (after intervention): 136 [120–160]		EG1 (after intervention: 61[48–78]		EG1 (after intervention): 97 [89–100]	
	EG2 (after intervention):135 [119–153]		EG2 (after intervention: 58 [45–82]		EG2 (after intervention): 98 [94–100]	
M. L. M. Santos et al. (2009)	NR	—	NR	—	NR	—
Tavares et al. (2019)	T1: 151 ± 16,49	0.006*	T1: 54.27 ± 9.01	0.374	T1: 97.73 ± 2.45	0.212
	T2: 156.57 ± 19.16		T2: 56.10 ± 12.43		T2: 98.27 ± 1.8	
	T3: 150.07 ± 17.78		T3: 54.37 ± 10.13		T3: 97.53 ± 2.9	
Wong and Fok (2003)	EG1: 9 [5–15]	0.07	NR	—	EG1: 0 [0–0]	0.2
	EG2: 14 [6–22]				EG2: 0 [0–0]	
Wong and Fok (2006)	NR	—	NR	—	NR	—

^a^ This study reported statistical significance without reporting the corresponding numerical results.

*p < 0.005.

Pain was evaluated in five studies using neonatal pain assessment scales (Oliveira et al. 2021; Martins et al. 2013; Moura et al. 2017; Oliveira et al. 2017; Tavares et al. 2019). Within the respective study populations, the authors reported either no significant changes or reductions in immediate pain scores following respiratory physiotherapy interventions.

Respiratory distress was assessed in three studies (Mehta et al. 2016; Oliveira et al. 2017; Tavares et al. 2019). The authors reported reductions in respiratory distress scores immediately after the interventions within their respective study samples. For example, Mehta et al. (2016) observed lower Silverman–Andersen scores during the immediate post‐intervention assessment; however, no longer‐term follow‐up was performed.

Lung mechanics were evaluated in two studies (M. L. M. Santos et al. 2009; Wong and Fok 2006). The authors reported improvements in respiratory system compliance after lung squeezing (Wong and Fok 2006) and after airway clearance techniques in mechanically ventilated neonates (M. L. M. Santos et al. 2009). One study also described reductions in airway resistance following the intervention (M. L. M. Santos et al. 2009). These findings represent study‐specific physiological observations and should not be interpreted as evidence of generalized clinical effectiveness.

A summary of the primary conclusions as stated by the authors of each included article is presented in Table 5.

**TABLE 5:** Conclusions of the studies included.

Author, year	Objective	Conclusion
Oliveira et al. (2021)	To verify if the techniques focusing on muscle synergy could cause immediate pain in neonates with transitory tachypnea.	Techniques focusing on muscle synergy does not influence in pain, and, when applied in a calm environment, reduced NIPS immediate evaluation score in neonates with transitory tachypnea, and within this specific sample, reductions in respiratory frequency and improvements in peripheral oxygen saturation were reported by the authors.
Diwate et al. (2018)	To find out the effectiveness of chest physiotherapy along with positioning and conventional chest physiotherapy in ventilated neonates.	Chest physiotherapy in the prone position for ventilated neonates was reported to result in higher oxygen saturation (SpO_2_) and partial pressure of oxygen in the arterial blood (PaO_2_) when compared to conventional chest physiotherapy alone within the studied pilot sample.
Guerra et al. (2017)	To evaluate thoracoabdominal mobility by photogrammetry in newborns after expiratory flow increase technique.	Expiratory flow increase technique in healthy newborns, when evaluated by biophotogrammetry, does not seem to change thoracoabdominal mobility.
Martins et al. (2013)	To analyze the effects of two different chest physiotherapy techniques in the occurrence of pain and in cardiorespiratory parameters in stable newborns.	Regarding the two chest physiotherapy protocols, patients within this trial did not present any significant changes in terms of pain and cardiorespiratory parameters evaluation.
Mehta et al. (2016)	To verify the positive and negative effects, if any at all, of chest physiotherapy maneuvers in preterm neonates.	The authors concluded that the evaluated protocol was tolerated without immediate adverse effects in their specific sample of preterm neonates. Airway suctioning was associated with immediate changes in cardiorespiratory parameters.
Moura et al. (2017)	To assess if the effects of the vibration and expiratory flow acceleration techniques might cause pain to preterm infants diagnosed with pneumonia.	Expiratory flow acceleration was reported to cause fewer changes over heart rate whist vibration technique was observed to have a lesser impact on facial mimetic aspects in preterms diagnosed with pneumonia. Neither one of the techniques was found to cause significant alterations within the studied sample.
Nicolau and Falcão (2010)	To assess the effects of respiratory physio therapy on cardiopulmonary function of ventilated preterm newborns infants.	Respiratory therapy and endotracheal suction were observed to have no significant influences on cardiopulmonary parameters in the evaluated cohort.
Oliveira et al. (2017)	To compare conventional respiratory physiotherapy and techniques focusing on muscle synergy in newborns with transient tachypnea of the newborn.	Within the limits of this trial, the authors described greater short‐term improvements in respiratory biomechanics, respiratory rate and respiratory distress, in addition to not causing pain in newborn infants with transient tachypnea of the newborn for the muscle synergy group compared to the conventional protocol.
M. L. M. Santos et al. (2009)	To evaluate the repercussions of specific physiotherapy techniques on the airway average resistance and lung dynamic compliance of preterm neonates on mechanical ventilation.	Airway clearance techniques were associated with short‐term modifications in average airway resistance and lung dynamic compliance in this specific cohort of preterm newborns on mechanical ventilation for ≥ 5 days.
Tavares et al. (2019)	To evaluate the occurrence of acute adverse physiological alterations and the presence of pain in preterm newborns with respiratory distress syndrome after chest physical therapy.	Physiological and behavioral parameters were reported to remain stable after chest physical therapy, with slight alterations immediately after the procedure, but with return to baseline values in a short period of time. Within the investigated cohort, chest physical therapy was not found to sharply alter vital signs and pain levels of newborns.
Wong and Fok (2003)	To compare the effectiveness and safety of using lung squeezing technique with conventional percussion and vibration protocol for correcting atelectasis in ventilated neonates.	There was no significant difference in hemodynamic disturbances between lung squeezing technique and conventional percussion and vibration protocol groups. Lung squeezing technique was described by the authors as more effective for re‐expansion of lung atelectasis within this specific investigated sample.
Wong and Fok (2006)	To investigate the effects of lung squeezing technique on the parameters of lung mechanics in preterm infants on mechanical ventilation.	Lung squeezing technique was reported to increase total respiratory system compliance immediately post‐intervention, possibly through decompression of the overdistended lung units and recruitment of atelectatic alveoli. Respiratory system resistance showed no significant change after lung squeezing technique.

**FIGURE 2:**
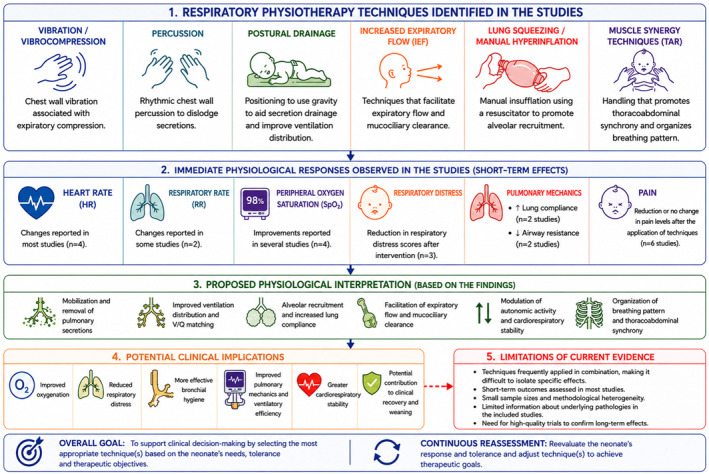
Structured clinical reasoning framework for neonatal respiratory physiotherapy based on the findings of the included studies. The framework summarizes the main respiratory physiotherapy techniques identified in the studies, the immediate physiological responses reported, the proposed physiological interpretations, potential clinical implications, and the current limitations of available evidence. The figure was developed based on the synthesis and analytical interpretation of the studies included in this scoping review, with graphical support generated using artificial intelligence (ChatGPT, OpenAI, San Francisco, CA, USA).

For a comprehensive overview of the synthesized findings, Figure 2 presents a conceptual framework mapping the physiotherapeutic techniques, reported physiological responses, and clinical implications identified in this review.

Overall, the mapped evidence demonstrated considerable heterogeneity regarding neonatal populations, intervention protocols, treatment frequency, comparator groups, and reported outcomes. Most studies evaluated immediate physiological responses after single‐session interventions, whereas clinically relevant outcomes and long‐term follow‐up were rarely investigated.

### 2.6 Discussion

This scoping review mapped the respiratory physiotherapy interventions evaluated in neonates admitted to NICUs and demonstrated that the available evidence remains highly heterogeneous regarding patient populations, intervention protocols, and reported outcomes. Therefore, the findings should be interpreted as a descriptive overview of current research rather than as evidence supporting the effectiveness or superiority of specific techniques. Although several studies reported short‐term changes in cardiorespiratory parameters following physiotherapy interventions, substantial variability in study designs and outcome measures precludes direct comparisons across techniques. Consequently, the findings synthesized in this scoping review—including the data mapped across the tables and figures—should be regarded primarily as a descriptive mapping of current clinical practices rather than as evidence supporting the effectiveness of any specific interventions. Any clinical interpretation regarding the efficacy, safety or superiority of particular techniques should be made with considerable caution and should not be interpreted as an endorsement, given the marked heterogeneity in neonatal populations (e.g., preterm vs. term infants, mechanically ventilated vs. spontaneously breathing neonates) and clinical settings.

Beyond mapping the available interventions, this review contributes to the existing literature by highlighting the heterogeneity of intervention protocols, the predominance of multimodal therapeutic approaches, and the emphasis on immediate physiological outcomes, all of which limit the development of more precise clinical recommendations. Furthermore, the review identified important methodological and conceptual gaps that may directly influence the interpretation and clinical applicability of respiratory physiotherapy interventions in neonatal care. Collectively, these findings underscore the need for a more structured clinical reasoning framework capable of integrating neonatal characteristics, respiratory conditions, physiological responses, and individualized therapeutic goals when selecting respiratory physiotherapy interventions.

Cardiorespiratory parameters, including heart rate, respiratory rate, and oxygen saturation, were the most frequently reported outcomes. These measures are commonly used in neonatal research because they provide rapid and objective indicators of physiological responses to therapeutic interventions (Corvaglia et al. 2020). However, the predominance of immediate physiological outcomes represents an important limitation of the current evidence base. Few studies assessed broader and more clinically meaningful outcomes, such as duration of respiratory support, length of hospital stay, or long‐term respiratory function.

This limitation is particularly relevant in a neonatal intensive care environment, where newborns are inherently vulnerable to cardiorespiratory instability and where clinical decision‐making requires consideration of both short‐ and long‐term outcomes. Such instability occurs during the first 72 hours of life (e.g., episodes of hypoxemia and bradycardia). For example, in a study involving 24 preterm infants with a gestational age of less than 32 weeks and/or birth weight under 1500 g, a total of 1050 cardiorespiratory events were recorded within the initial 72‐h period (Corvaglia et al. 2020). This instability may be related to lower gestational age, lower Apgar scores, the presence of congenital heart disease, incidence of neurological insults, and even adverse effects of medications (Corvaglia et al. 2020; Corvaglia et al. 2020).

Although these physiological parameters are clinically valuable for assessing the acute tolerance and safety of interventions, they provide limited insight into functional recovery, respiratory progression, or long‐term pulmonary health. Moreover, isolated short‐term physiological improvements do not necessarily translate into clinically meaningful benefits, such as reduced duration of ventilatory support, lower incidence of pulmonary complications, shorter hospital stays, or improved long‐term respiratory outcomes. Future studies should therefore prioritize clinically relevant endpoints, including duration of respiratory support, length of hospitalization, feeding tolerance, and long‐term respiratory follow‐up. The inclusion of functional and long‐term outcomes would provide a more comprehensive understanding of the true clinical impact of respiratory physiotherapy interventions in neonates and help determine whether the immediate physiological changes observed following treatment translate into meaningful clinical benefits.

Although improvements in physiological parameters such as heart rate, respiratory rate, and oxygen saturation are frequently observed following respiratory physiotherapy interventions, these findings primarily reflect the safety and tolerability of the treatment rather than its clinical effectiveness. The scarcity of studies evaluating clinically relevant outcomes, such as duration of mechanical ventilation, length of hospital stay, and long‐term respiratory outcomes, limits the understanding of the true impact of these interventions on neonatal recovery (Oliveira et al. 2021; Diwate et al. 2018; Guerra et al. 2017; Martins et al. 2013; Mehta et al. 2016; Moura et al. 2017; Nicolau and Falcão 2010; Oliveira et al. 2017; M. L. M. Santos et al. 2009; Tavares et al. 2019; Wong and Fok 2003, 2006).

Considerable variability was also observed in the characteristics of the neonatal populations included in the studies, which involved both preterm and term infants with different respiratory conditions. These differences are clinically significant because gestational age, pulmonary maturity, respiratory mechanics, and underlying respiratory conditions may substantially influence both physiological responses and tolerance to respiratory physiotherapy interventions. Preterm neonates, particularly those born at extremely low gestational ages, exhibit increased respiratory vulnerability due to pulmonary immaturity and cardiorespiratory instability. In contrast, term neonates may require different therapeutic approaches and may demonstrate distinct response patterns depending on the underlying clinical condition (Rodríguez‐Roza et al. 2026). Therefore, the heterogeneity of neonatal populations included in the available studies limits the generalizability of the findings and highlights the need for future investigations focused on more homogeneous clinical subgroups.

An overall analysis of the different interventions, neonatal subgroups, and outcome measures revealed consistent patterns across the included studies. Conventional respiratory physiotherapy techniques, such as vibrocompression, chest percussion, postural drainage, and expiratory flow acceleration, were more frequently associated with immediate effects on cardiorespiratory and pulmonary mechanics parameters. In contrast, interventions based on positioning and muscle synergy approaches were more closely related to the promotion of respiratory stability and ventilatory adaptation. Furthermore, the outcomes assessed varied according to the clinical characteristics of the neonatal populations. Studies involving more vulnerable preterm infants primarily focused on physiological parameters, whereas those including clinically stable newborns also incorporated assessments of treatment tolerance and recovery. Overall, improvements in pulmonary mechanics, oxygenation, and cardiorespiratory stability were observed simultaneously, suggesting the involvement of interdependent physiological mechanisms. Nevertheless, the substantial heterogeneity in interventions, patient populations, and outcome measures limits the identification of the most effective strategies for specific neonatal subgroups and distinct clinical objectives (M. L. M. Santos et al. 2009; Wong and Fok 2006).

In addition, intervention protocols varied substantially in the techniques used, session duration, and treatment frequency. In several studies, multiple physiotherapy techniques were applied during the same session, making it difficult to determine the contribution of individual maneuvers to the reported outcomes. This methodological diversity limits the reproducibility and comparability of findings across studies and reduces the ability to establish evidence‐based recommendations regarding the effectiveness of specific respiratory physiotherapy techniques. From a clinical perspective, although multimodal interventions may more accurately reflect routine practice in neonatal intensive care units, the absence of standardized and isolated intervention protocols makes it difficult to determine the specific physiological effects and safety profile of isolated techniques. Future randomized controlled trials should therefore prioritize standardized intervention protocols and investigate isolated physiotherapeutic strategies to better elucidate their individual contributions to neonatal respiratory outcomes.

Because of their increased vulnerability to cardiorespiratory instability, these patients require greater care from the respiratory physiotherapy service. Although some studies suggest that certain respiratory physiotherapy techniques caused clinical instability in these newborns (Ambalavanan et al. 2023; Nicolau and Lahóz 2007), several studies reported short‐term changes in cardiorespiratory parameters after respiratory physiotherapy sessions; however, these findings should be interpreted cautiously due to the heterogeneity of interventions and study designs (Oliveira et al. 2021; Tavares et al. 2019). The study by Martins et al. (2017), which investigated the effects of respiratory physiotherapy on pain and cardiorespiratory function in 60 newborns at three different time points, found no significant changes in any cardiorespiratory parameters. In the study by Nicolau and Falcão (2010), which evaluated a total of 252 physiotherapy sessions conducted on 42 preterm infants, it was demonstrated that heart rate, respiratory rate, peripheral oxygen saturation, and respiratory pattern remained within physiological limits after the procedures, suggesting that, when performed appropriately, they do not compromise the clinical stability of these patients.

It is important to emphasize that, although combined interventions may better reflect the complexity of real‐world neonatal practice, they also complicate the identification of the specific therapeutic components responsible for the observed physiological responses. Combinations of positioning strategies, tactile stimulation, vibrocompression, and bronchial hygiene techniques may exert synergistic effects on ventilation–perfusion matching, secretion clearance, respiratory mechanics, and autonomic regulation (M. L. M. Santos et al. 2009). However, the scarcity of studies evaluating isolated interventions limits the understanding of the individual physiological contributions of each technique and hinders the development of more targeted therapeutic protocols. These findings underscore the importance of future research specifically designed to isolate individual components of respiratory physiotherapy and clarify their mechanisms of action across different neonatal populations.

Most studies reported no significant adverse physiological responses during or immediately after the interventions. However, safety outcomes were inconsistently reported, and the available evidence remains limited. Therefore, caution is warranted when interpreting the findings and translating them into clinical practice. Some studies (Oliveira et al. 2021; Martins et al. 2013; Moura et al. 2017; Oliveira et al. 2017; Tavares et al. 2019) evaluated the presence of pain after respiratory physiotherapy techniques. The studies by Martins et al. (2013) and Moura et al. (2017) showed that respiratory physiotherapy did not alter the pain patterns assessed in hospitalized newborns. On the other hand, a review study (Zanelat et al. 2017), where more than 400 newborns were evaluated, found the presence of pain during manual vibration, vibrocompression, and airway suction techniques, a result similar to that reported by Tavares et al. (2019), included in this review, where there was a slight increase in pain scores immediately after thoracic vibration and airway suction techniques.

Airway suction is a painful procedure (Gardner and Shirland 2009; Cignacco et al. 2008), and although it is part of the routine care of the physiotherapy service, it cannot be considered exclusive to this professional category. Painful stimuli not only alter behavioral patterns but also a range of physiological parameters in neonates beyond pain, such as variations in cerebral blood flow, heart rate, and SpO2 (Gardner and Shirland 2009; Cignacco et al. 2008; Silva et al. 2007). Changes in these parameters were also evidenced in the study by Mehta et al. (2016), which, although it did not assess pain in its sample, found a significant impact on heart rate and oxygen saturation after the airway suction procedure.

The studies by Santos et al. (M. L. M. Santos et al. 2009) and Wong and Fok (Wong and Fok 2006) evaluated aspects of respiratory mechanics, including lung compliance and resistance, following respiratory physiotherapy. In both studies, an increase in lung compliance was observed. Regarding lung resistance, Santos et al. (M. L. M. Santos et al. 2009) reported a significant reduction in this variable in both studied groups. These results are consistent with the study by Rosa et al. (2007), conducted in adults undergoing mechanical ventilation, which evaluated the effects of bronchial hygiene techniques on respiratory mechanics and demonstrated that their application improved average airway resistance but had little effect on dynamic compliance. A robust systematic review (Igual et al. 2023) evaluated the effects of respiratory physiotherapy in preterm infants with Respiratory Distress Syndrome and found positive results, including increased respiratory system compliance and reduced inspiratory and expiratory resistance.

Although the findings mapped in this review suggest a potential physiological association between respiratory physiotherapy interventions and modulation of pulmonary mechanics, such interpretations are derived from highly specific and heterogeneous clinical contexts. Therefore, these observations should be viewed as hypotheses‐generating rather than as definitive clinical evidence. Potential mechanisms include optimization of lung expansion, reduction of airway obstruction, improvement of thoracoabdominal synchrony, and facilitation of airflow distribution. Nevertheless, the methodological limitations and heterogeneity of the included studies preclude definitive conclusions regarding the superiority of individual techniques or the formulation of specific clinical recommendations.

The wide variation in nomenclature and therapeutic protocols observed in our scoping review mirrors a broader, ongoing challenge in respiratory care. As recently highlighted by Reychler et al. (2026), there is an urgent international need to standardize airway clearance techniques based strictly on their underlying physical definitions and physiological mechanisms rather than traditional, ambiguous terminology. By mapping these techniques and identifying these gaps, this review aligns with contemporary efforts to transition toward mechanism‐based interventions in neonatal care.

Some studies included in this scoping review (Oliveira et al. 2021; Mehta et al. 2016; M. L. M. Santos et al. 2009; Wong and Fok 2003, 2006) investigated the effects of chest physiotherapy techniques in mechanically ventilated newborns and reported improvements in several physiological parameters; however, these findings are mostly derived from small, heterogeneous studies. These findings are consistent with other research demonstrating favorable outcomes of respiratory therapy in babies undergoing mechanical ventilation, including improved SpO_2_ and respiratory rate, as well as reduced overall mechanical ventilation duration (Macedo et al. 2024; Tana et al. 2023).

Although the available findings are encouraging, most included studies were characterized by small sample sizes, and relatively few randomized controlled trials were identified, substantially limiting the strength and generalizability of the evidence. Consequently, while respiratory physiotherapy appears to be feasible and generally safe in neonatal intensive care settings, the current evidence remains insufficient to support robust recommendations regarding the optimal techniques, treatment frequency, or duration of intervention. Furthermore, the methodological quality of the included studies was variable, with important limitations related to participant selection, lack of blinding, inadequate reporting of allocation procedures, and substantial heterogeneity in intervention protocols and outcome measures. These limitations increase the risk of bias and should be carefully considered when interpreting the physiological and clinical effects of respiratory physiotherapy interventions in neonates.

These methodological limitations have important implications for the interpretation of the available evidence. The predominance of studies with small sample sizes reduces statistical power and consequently increases the likelihood that the observed findings may be influenced by random variation or population‐specific characteristics. Furthermore, the scarcity of randomized controlled trials limits the ability to establish causal relationships between respiratory physiotherapy interventions and the outcomes achieved. Another important consideration is that many studies focused exclusively on the immediate effects of a single treatment session, which may not accurately reflect the cumulative impact of respiratory physiotherapy as it is routinely implemented in clinical practice. Therefore, although several studies have reported favorable physiological responses, the overall certainty of the evidence remains limited, and the findings should not be regarded as conclusive proof of clinical effectiveness but rather as indicators of potential beneficial effects (Oliveira et al. 2021; Diwate et al. 2018; Guerra et al. 2017; Martins et al. 2013; Mehta et al. 2016; Moura et al. 2017; Nicolau and Falcão 2010; Oliveira et al. 2017; M. L. M. Santos et al. 2009; Tavares et al. 2019; Wong and Fok 2003, 2006).

This scoping review highlighted a considerable scarcity of randomized controlled trials addressing the effects of respiratory physiotherapy techniques in newborns. Furthermore, as the techniques are generally applied in combination with other interventions, it becomes difficult to assess the specific effects of each maneuver on the investigated outcomes. It is also noteworthy that most studies do not provide detailed information about the pathologies affecting these newborns at the time respiratory techniques are applied, which might reduce the value of their findings and conclusions.

Overall, the available evidence suggests that respiratory physiotherapy interventions may produce multidimensional effects, encompassing not only physiological but also mechanical and clinical domains. Although improvements in oxygen saturation, respiratory rate, lung compliance, and airway resistance were among the most frequently reported findings, these outcomes should not be interpreted in isolation. Changes in pulmonary mechanics may contribute to enhanced gas exchange and cardiorespiratory stability, while reductions in respiratory workload may indirectly improve neonatal comfort and tolerance to care procedures. This holistic perspective reinforces the importance of considering neonatal respiratory physiotherapy within a broader framework that takes into account individual patient characteristics and respects the specific needs of each neonate, as well as underlying respiratory conditions and therapeutic goals, rather than focusing exclusively on isolated physiological outcomes (M. L. M. Santos et al. 2009; Wong and Fok 2006; Rosa et al. 2007; Igual et al. 2023; Macedo et al. 2024).

In addition to mapping the existing evidence, this review also highlights several important gaps in the literature on respiratory physiotherapy in neonates. First, there are few well‐designed randomized controlled trials evaluating these interventions, and many studies rely on small samples and heterogeneous study designs. Second, most studies assessed only short‐term responses following single physiotherapy sessions, leaving the cumulative and long‐term effects of these interventions largely unexplored. Third, several studies did not clearly describe the clinical conditions of the newborns at the time the interventions were applied, which limits the interpretation of the results and the identification of the most appropriate techniques for specific respiratory conditions. Additionally, many studies evaluated combinations of techniques rather than isolated interventions, making it difficult to determine the specific contribution of each maneuver. Finally, extremely preterm infants remain underrepresented in the available literature, despite being a particularly vulnerable population.

To address these critical gaps with greater specificity, future research must move toward methodologically rigorous frameworks across five main points. First, standardized intervention protocols should be established based on precise physical and physiological mechanisms rather than ambiguous nomenclature, explicitly defining and reporting the frequency (e.g., twice daily), total session duration (e.g., strictly 10 or 15 minutes), and the specific manual forces or positioning sequences utilized. Second, consensus is urgently needed regarding a core outcome set (COS) for neonatal respiratory care; this should systematically combine immediate safety and tolerability indicators (utilizing validated, multi‐dimensional tools such as the Premature Infant Pain Profile [PIPP] and Silverman‐Andersen score) with broader functional outcomes, such as ventilation‐free days, time to successful extubation, and feeding tolerance. Third, future trials must implement strict subgroup stratification to isolate therapeutic effects across distinct clinical phenotypes, separating neonates by gestational age (e.g., extremely preterm [< 28 weeks] vs. late preterm and term), respiratory support modality (invasive mechanical ventilation vs. non‐invasive support), and primary underlying lung pathology (e.g., Respiratory Distress Syndrome, transient tachypnea, or atelectasis). Fourth, studies should expand beyond immediate, short‐term evaluations to include longer follow‐up periods, monitoring cumulative physiological responses at 24 and 48 hours post‐intervention, as well as tracking outcomes up to NICU discharge. Finally, the priority for future randomized controlled trials (RCTs) should encompass multi‐center, parallel‐group designs that evaluate isolated, single‐maneuver strategies against a standardized control protocol, ensuring robust allocation concealment and independent outcome blinding to minimize the risk of bias.

An important limitation of the available evidence identified in this review is that most included studies involved short interventions, many consisting of only a single session of respiratory physiotherapy. Additionally, small sample sizes were observed in a significant proportion of studies, and no patient follow‐up was conducted to assess the long‐term effects of these interventions. Furthermore, several studies did not clearly describe the clinical criteria used to indicate the need for respiratory physiotherapy. Another relevant limitation is that only two of the included studies involved preterm infants with a gestational age below 29 weeks. Although this population is particularly vulnerable, the two studies by Wong and Fok (2003, 2006) did not report adverse effects associated with the interventions. Earlier studies on respiratory physiotherapy, particularly those from the late 1980s and early 1990s, reported adverse effects in extremely preterm infants when interventions were not appropriately structured or monitored. However, the studies analyzed here employed clinically appropriate and gentle techniques, with no complications observed. Future research should prioritize well‐designed randomized clinical trials with larger samples of newborns, clearer characterization of clinical conditions, and longer intervention and follow‐up periods.

### 2.7 Implications of Physiotherapy Practice

The included studies described short‐term changes in cardiorespiratory parameters without consistent reports of increased pain.

This scoping review mapped the respiratory physiotherapy interventions investigated in neonates admitted to neonatal intensive care units (NICUs) and summarized the outcomes reported across clinical studies. The available evidence remains limited and highly heterogeneous regarding neonatal populations, intervention protocols, and outcome measures. Consequently, the findings should be interpreted as a descriptive overview of current evidence rather than confirmation of the effectiveness, safety, or superiority of specific respiratory physiotherapy techniques.

Most studies evaluated multimodal interventions applied during a single treatment session, limiting comparisons across studies and making it difficult to determine the individual contribution of specific respiratory physiotherapy techniques. In addition, the included populations varied considerably with respect to gestational age, respiratory conditions, and ventilatory support, suggesting that differences in reported outcomes are likely influenced by patient characteristics and therapeutic objectives as much as by the interventions themselves.

Cardiorespiratory parameters were the most frequently reported outcomes and provide useful information regarding the immediate physiological response to respiratory physiotherapy. However, most studies assessed only short‐term physiological changes, whereas clinically relevant outcomes, including duration of respiratory support, length of hospital stay, feeding tolerance, and long‐term respiratory function, were rarely investigated. Therefore, improvements in physiological variables should be interpreted as indicators of treatment tolerance rather than as evidence of clinical effectiveness.

Safety remains an important consideration in this population. Although most studies did not report clinically relevant adverse physiological responses, safety outcomes were inconsistently assessed and frequently accompanied by airway suctioning, a procedure known to induce pain and physiological instability independently of respiratory physiotherapy (Zanelat et al. 2017; Gardner and Shirland 2009; Cignacco et al. 2008; Silva et al. 2007). Consequently, the available evidence remains insufficient to establish the safety profile of individual respiratory physiotherapy techniques. Likewise, the few studies evaluating respiratory mechanics reported improvements in lung compliance and airway resistance, but these findings derive from small heterogeneous populations and should be regarded as hypothesis‐generating rather than definitive evidence of clinical benefit. Similar considerations apply to mechanically ventilated neonates, for whom encouraging physiological responses have been described but robust clinical evidence remains limited.

The methodological limitations of the available literature substantially influence the interpretation of these findings. Most studies included small sample sizes, few randomized controlled trials, heterogeneous intervention protocols, and predominantly single‐session interventions. In addition, important methodological limitations—including limited reporting of allocation procedures, blinding, and neonatal clinical characteristics—reduce the certainty and generalizability of the available evidence. Furthermore, extremely preterm infants remain underrepresented despite being one of the populations most likely to benefit from optimized respiratory physiotherapy strategies.

These findings highlight important priorities for future research. Standardized intervention protocols, harmonized terminology, and clinically meaningful core outcome measures are needed to improve comparability across studies. Future multicenter randomized controlled trials should evaluate isolated respiratory physiotherapy techniques, incorporate longer follow‐up periods, and stratify participants according to gestational age, respiratory support modality, and underlying pulmonary disease to better identify which interventions are most appropriate for specific neonatal populations.

Overall, this review demonstrates that respiratory physiotherapy in neonatal intensive care remains an evolving field characterized by substantial methodological and clinical heterogeneity. Current evidence primarily describes immediate physiological responses and provides an important overview of existing clinical practice but remains insufficient to support definitive recommendations regarding the effectiveness or safety of individual respiratory physiotherapy techniques. High‐quality studies are required to strengthen the evidence base and inform clinical decision‐making in neonatal respiratory care.

## Funding

The authors received financial support for the Coordination of Superior Level Staff Improvement (CAPES), Grant/Award Number: 001.

## Ethics Statement

The authors have nothing to report.

## Consent

The authors have nothing to report.

## Conflicts of Interest

The authors declare no conflicts of interest.

## Supporting information


Supporting Information S1

## Data Availability

The datasets generated and/or analyzed during the current study are available from the corresponding author upon request. (1) Access will be granted to qualified researchers for the purpose of secondary analysis, provided the request is accompanied by a formal research protocol and evidence of institutional ethics committee approval. (2) Access will not be granted for commercial purposes or to individuals without a verified institutional affiliation. (3) A formal Data Use Agreement (DUA) must be signed to ensure the protection of participant confidentiality and adherence to ethical standards. (4) Persistent contact for data requests: silvana.alves@ufrn.br.
