# Supplementary material for: Respiratory Physiotherapy Techniques in Neonatal Intensive Care Units: A Scoping Review
Source: Physiother Res Int. 2026 Aug 1;31(4):e70304. doi: 10.1002/pri.70304 (PMC13428499; doi:10.1002/pri.70304)
Supplement: Supplementary file 1 — Supporting Information S1 [file PRI-31-e70304-s001.docx]

Supplementary File 1 – Search strategies for each database

**Pubmed:** ((Newborn OR Newborns OR Newborn Infant OR Newborn Infants OR Neonate OR Neonates) AND (Chest Physical Therapy OR Conventional Chest Physical Therapy OR Chest Physiotherapy OR Physiotherapy Respiratory OR Physiotherapy OR Autogenic Drainage OR Expiratory Flow Increase Technique OR Prolonged Slow Expiratory Maneuver OR Technique of Re-educating Thoracic and Abdominal OR Chest Percussion OR Shaking OR Tapping) AND (Clinical Trials as Topic OR Clinical Trial as Topic OR Clinical Trial OR Random OR Random Allocation OR Randomization OR Therapeutic Use OR Therapeutic Uses OR Therapeutic Effects OR Therapeutic Effect OR Interventional Study OR Interventional Studies)).

**Cochrane Library:** ((Newborn OR Newborns OR Newborn Infant OR Newborn Infants OR Neonate OR Neonates) AND (Chest Physical Therapy OR Conventional Chest Physical Therapy OR Chest Physiotherapy OR Physiotherapy Respiratory OR Physiotherapy OR Autogenic Drainage OR Expiratory Flow Increase Technique OR Prolonged Slow Expiratory Maneuver OR Technique of Re-educating Thoracic and Abdominal OR Chest Percussion OR Shaking OR Tapping) AND (Clinical Trials as Topic OR Clinical Trial as Topic OR Clinical Trial OR Random OR Random Allocation OR Randomization OR Therapeutic Use OR Therapeutic Uses OR Therapeutic Effects OR Therapeutic Effect OR Interventional Study OR Interventional Studies)).

**Lilacs:** ((Newborn OR Newborns OR Newborn Infant OR Newborn Infants OR Neonate OR Neonates) AND (Chest Physical Therapy OR Conventional Chest Physical Therapy OR Chest Physiotherapy OR Physiotherapy Respiratory OR Physiotherapy OR Autogenic Drainage OR Expiratory Flow Increase Technique OR Prolonged Slow Expiratory Maneuver OR Technique of Re-educating Thoracic and Abdominal OR Chest Percussion OR Shaking OR Tapping) AND (Clinical Trials as Topic OR Clinical Trial as Topic OR Clinical Trial OR Random OR Random Allocation OR Randomization OR Therapeutic Use OR Therapeutic Uses OR Therapeutic Effects OR Therapeutic Effect OR Interventional Study OR Interventional Studies)).

**Web of Science:** ((Newborn OR Newborns OR Newborn Infant OR Newborn Infants OR Neonate OR Neonates) AND (Chest Physical Therapy OR Conventional Chest Physical Therapy OR Chest Physiotherapy OR Physiotherapy Respiratory OR Physiotherapy OR Autogenic Drainage OR Expiratory Flow Increase Technique OR Prolonged Slow Expiratory Maneuver OR Technique of Re-educating Thoracic and Abdominal OR Chest Percussion OR Shaking OR Tapping) AND (Clinical Trials as Topic OR Clinical Trial as Topic OR Clinical Trial OR Random OR Random Allocation OR Randomization OR Therapeutic Use OR Therapeutic Uses OR Therapeutic Effects OR Therapeutic Effect OR Interventional Study OR Interventional Studies)).

**SciELO:** ((Newborn) AND (Physical Therapy Modalities OR Physical Therapy Specialty OR Respiratory Therapy)).

**Science Direct:** ((Newborn OR Neonate) AND (Chest Physiotherapy OR Physiotherapy Respiratory) AND (Clinical Trial as Topic OR Random Allocation OR Therapeutic Use OR Therapeutic Effect OR Interventional Study)).
